# The Medical Student’s Guide in Extracting Teeth; with Numerous Cases in the Surgical Branch of Dentistry

**Published:** 1851-05

**Authors:** 


Art. VI.—*The Medical Student's Guide in Extracting Teeth;* with numerous cases in the surgical branch of Dentistry. With illustrations. By S. S. Hornor, Practical Dentist. Philadelphia: Lindsay & Blakiston. 1851.

Mr. Hornor has prepared an excellent manual, on a subject which is not generally studied enough in preparing for the practice of medicine. Students rush in crowds to see a formidable operation in surgery, and treat with indifference those matters that may make daily calls upon them. Among these matters is the treatment of toothache, and it is important that a physician shall know all he can possibly learn on such subjects. He should qualify himself in all his responsible duties well enough to discharge them with ease to the sufferers and credit to his science; and a man's qualifications for other portions of his profession may be judged from his performance of as small a duty as the extraction of a tooth. There is a large volume of medical science connected with carious teeth, and we do not see how a medical practitioner can pretend to understand his profession, or offer his services as a physician, without an intimate knowledge of everything connected with diseased conditions of the teeth. In numerous cases an accurate diagnosis depends upon this knowledge, and by it he is led to form true views of what would be a field of error without it. There is no more reason why a carious tooth should be driven from the management of the medical practitioner, than a carious tibia, or femur, and it is often as important to understand the first as either of the two last.

Mr. Hornor has written his little guide on the extraction of teeth with a thorough appreciation of his subject, and his rules and advice are very excellent. The instruments he commends "as absolutely necessary, are, the straight forceps, the double curved forceps, the key, the elevator, punch, and screw; the curved forceps, the hawkbill, and the wedge forceps, several of which are applicable to the jaws."

There is a concise and clear explanation in this little work of the uses of the various instruments, we have enumerated, in the proper application to the various teeth, and we advise inexperienced practitioners in tooth drawing to make this short treatise the man of their counsel on the extraction of teeth. Such persons will never have cause to regret a careful perusal of this work, and some of their patients may have good cause for feeling grateful to them for having condescended to study Hornor on tooth drawing.

The admirable little monograph before us is well printed, and the illustrative drawings of the instruments are admirably executed. By the proper use of proper instruments, the extraction of teeth may require a smaller degree of anasthesia than the awkward bungler finds necessary. B.

